# Reasoning supports forgiving accidental harms

**DOI:** 10.1038/s41598-021-93908-z

**Published:** 2021-07-13

**Authors:** Indrajeet Patil, Bastien Trémolière

**Affiliations:** 1 Center for Humans and Machines, Max Planck Institute for Human Development, Berlin, Germany; 2 APSY-V, University of Nîmes, 30021 Nîmes Cedex 1, France

**Keywords:** Psychology, Human behaviour

## Abstract

People experience a strong conflict while evaluating actors who unintentionally harmed someone—her innocent intention exonerating her, while the harmful outcome incriminating her. Different people solve this conflict differently, suggesting the presence of dispositional moderators of the way the conflict is processed. In the present research, we explore how reasoning ability and cognitive style relate to how people choose to resolve this conflict and judge accidental harms. We conducted three studies in which we utilized varied reasoning measures and populations. The results showed that individual differences in reasoning ability and cognitive style predicted severity of judgments in fictitious accidental harms scenarios, with better reasoners being less harsh in their judgments. Internal meta-analysis confirmed that this effect was robust only for accidental harms. We discuss the importance of individual differences in reasoning ability in the assessment of accidental harms.

## Introduction

In 2010, the rock band *Lamb of God* was performing in Czech Republic and, during the performance, the lead singer Randy Blythe threw a fan named Daniel Nosek off the stage, with the expectation that other people will catch him. Nosek instead fell backwards directly on his head, suffered severe traumatic brain injury, slipped into a coma, and died weeks later from his injuries. If we were to be part of the jury who was going to decide how morally bad Blythe’s behavior was and how much we should punish him, how would we go about it? Will we focus on his innocent intentions and reasonable beliefs about how things should have unfolded? Or will we be swayed by a strong emotional reaction in response to the details about suffering that Nosek had to endure because of Blythe’s actions? Will deliberating about the situation help us subdue influence of this emotional reaction on our decisions?

When it comes to evaluating third-party harmful behavior like this, past work has shown that people rely not only on the assessment of the mental state of the perpetrator, but also on the presence of a harmful consequence for the victim^1–7^. In other words, after witnessing a harmful event, a third-party moral judge reasons about the actor’s intentions (“What was Blythe *thinking* when he threw his fan off the stage?!”) and the victim’s feelings (“How *painful* it must have been for Nosek to suffer a head trauma?”). Not only has the past work validated this two-part template of intent-based morality at the psychological level, but also explored the neural substrates for these two independent processes^1^. In particular, this work reveals that the observers decode intentional status of interpersonal harmful actions via a network of brain regions—known as the Theory of Mind network—involved in representing others’ thoughts^8^, while representing victim’s feeling states recruits the “empathy for pain” network^9^. Probably the most salient way to demonstrate the dissociable contributions of these two processes towards moral evaluations is by focusing on how people judge accidents. Accidental harms elicit a strong conflict in the observer/judge because the two processes conflict with each other in terms of their output: the intent-based process focuses on innocent intentions of the actor and *reduces* severity of moral evaluations^10,11^, while the outcome-based process localizes on empathic reaction towards the victim suffering and the agent’s causal role in producing this outcome and *increases* severity of moral condemnation^9^. As a result, how we judge accidents depends on how we resolve the conflict posed by these two processes: difficulties in processing intentions leads to more punitive attitudes (e.g., autistic individuals^12^), while deficits in empathic reaction towards the victim can lead to forgiving attitudes (e.g., psychopathy and sadism^13,14^). In other words, forgiving accidental harms *feels* so difficult because it involves overriding a potent emotional reaction to victim suffering with a more deliberative response stemming from reasoning about intentionality. It is worth noting that this conflict is specific to accidents and is *not* encountered while evaluating other interpersonal interactions. When the actor *intentionally* harms someone, two processes agree on condemnation^15^, while when the actor *attempts* to harm someone but fails, there is no strong empathic emotional response that needs to be counteracted by the mentalizing system and the two process again agree on condemnation^1,8,9,16^.

Although past work has thoroughly explored the processes that give rise to conflict while pondering over accidents and the role of dispositional mentalizing and empathizing abilities in resolving this conflict, much less attention has been paid to how one’s ability and willingness to engage in analytical reasoning affect this conflict resolution. Indeed, a hint for such a role comes from work with sacrificial moral dilemmas. Sacrificial moral dilemmas, like accidents, pose a conflict of a different variety—between the emotionally aversive “utilitarian” option of personally harming someone and the option of letting a greater number of individuals get hurt. Past work shows that differences in individual’s ability to reason and availability of cognitive resources play a role in how this conflict is resolved (for a review, see^17–19^). Current work is inspired by these prior investigations.

A decade long research in the field of moral psychology has revealed how our moral judgments are, at the broadest level, the result of an interplay between emotions on the one hand, and reason on the other^20–22^. Although there are many variants of dual process models^21,23–26^, the generic version distinguishes a fast, parallel, and almost automatic thinking system (*intuitive system*) from a slow, sequential, and cognitively effortful thinking system (*analytical system*). Note that reasoning psychologists use words interchangeably to define the two systems (e.g., intuitive/heuristic system versus analytical/deliberative/reflective system). For the sake of consistency, we will always use the words *intuitive* system/participant vs. *analytical* system/participant.

Considering that the human mind is composed of two thinking systems has led researchers to design specific manipulations and measures to test predictions derived from the dual-process model, and these protocols have proven to be useful in the field of moral judgment and decision making^17^. Specifically, numerous tasks have been designed that typically make an intuitive response conflict with an analytical response, and numerous measures have been used to capture the underlying psychological mechanisms (e.g. response time measure, time pressure manipulation, interfering cognitive load manipulation, interindividual differences, etc.)^17^. The utility of this corpus of measures and manipulations has been thoroughly explored in the context of sacrificial moral dilemmas^27–38^. It is also worth noting, however, that there are studies which have found no effects of some of these manipulations on moral judgment activities^39^, or which reanalyze previous work to highlight their limited generalizability^40,41^. Additionally, the “corrective” dual-process model, which posits correction of the “intuitive” response by “deliberative” processes, has also been called into question^42–45^. Current work focuses only on *interindividual differences* and is agnostic with regards to the time course or “intuitiveness” of the responses from two systems and therefore we will not discuss debate any further.

The starting point for us is prior work which argues that people who score higher on self-report or performance measures of reflective reasoning also tend to be more “utilitarian”^36^ while resolving sacrificial dilemma conflicts. In the current work, we plan to extend this work to explore more broadly the role of analytical reasoning in resolving the kind of conflict one encounters while evaluating behavior of unintentional harm-doers. In a manner reminiscent of how reasoning bolsters “utilitarian” inclinations on sacrificial dilemmas, we predict that more capable reasoners or people prone to analytical reasoning will resolve it by overriding the strong emotional response, which would lead to a greater acceptability of accidental harms.

## General methods

Across all studies, experimental stimuli consisted of intent-based moral vignettes^46^ that were result of a 2 × 2 within-subjects design where the factors *belief* (neutral, negative) and *outcome* (neutral, negative) were independently varied such that agents in the scenario produced either a neutral outcome or a harmful outcome while acting with the belief that they were causing either a neutral outcome or a harmful outcome. The magnitude of harm severity varied freely across scenarios from mild to severe to fatal injuries. We provide below an example of the parametric variation in a single scenario context in accidental harm condition:

*Background*

Matilda is walking by a neighbour’s swimming pool when she sees a child about to dive in.

*Foreshadow*

The child is about to dive into the shallow end and smack his head very hard on the concrete bottom of the pool.

*Belief*

Because of a label on the side of the pool, Matilda believes that the child is about to dive safely into the deep end and swim around.

*Outcome*

Matilda walks by, without saying anything to the child. The child dives in and breaks his neck.

The number of scenarios used and the type of question asked varied across studies, along with the scale used for measuring a response. These differences across studies are tabulated in Table 1. Additionally, detailed text for the scenarios are reproduced in Supplementary Text S2.

**Table 1:** Details about experimental designs included in Studies 1–3 and demographic details for participants.

Study	1	2	3		
Platform used	Lab-based	Lab-based	MTurk		
Sample size	44	112	1223	1212	
Average age	23.01	24.12	36.8	36.2	
Gender composition (% female)	66%	62.50%	50.12%	52.56%	
Country	Italy	Italy	USA		
Measure/manipulation	NFC	CRT (6-item)	REI	AOT	BB
Cronbach's alpha	0.873	–	0.940	0.740	0.660
Number of conditions	4	4	4		
Number of stories per participant	36	36	4		
Total number of stories used	144 (4 × 36)	144 (4 × 36)	16 (4 × 4)		
Number of data points (after exclusion)	3052	7813	4892	4848	
Type of ratings and their assignment	Acceptability and blame; within-subject	Acceptability and punishment; within-subject	Wrongness and punishment; between-subjects		
Scale	Likert	VAS	Likert		
Range of ratings	1–7	0–20	1–7		

Questions and scale labels used for different questions: Wrongness (How wrong was [the agent]'s behavior?; 1: *not at all*, 7: *very much*), Punishment (How much should [the agent]'s be punished?; 1 (or 0): *none at all*, 7 (or 20): *a lot*), Acceptability (“How morally acceptable was [the agent]’s behavior?”; 1 (or 0): *not at all acceptable*; 7 (or 20): *completely acceptable*), Blame: “How much blame does [the agent] deserve?” (1: *none at all*, 7: *very much*).

*AOT* actively open-minded thinking, *BB* belief bias, *CRT* cognitive reflection test, *NFC* need for cognition, *REI* rational-experiential inventory, *VAS* visual analog scale.

### Sampling stopping rule and exclusion criteria

These studies were each part of prior unrelated data collection, thus impeding any sampling size control in the present research.

For Amazon Mechanical Turk studies, following exclusion criteria were applied to leave out participants who: did not complete the entire survey, reported to be less than 18 years old or more than 100 years old, failed attention checks, completed the same survey multiple times. Additionally, we used TurkPrime to make the survey available for completion only to MTurk workers who had a rating of above 95% and had completed at least 100 other HITs. All sample sizes reported below refer to the *final* sample after these exclusion criteria had been applied.

### Data analysis

Since the behavioral data (items within conditions within participants) had multilevel or nested structure, we utilized mixed-effects models to correctly handle the inherent dependencies in nested designs and to reduce probability of Type I error due to reduced effective sample size^47–49^.

When null hypothesis significance testing (NHST) results in a failure to reject the null hypothesis (H0), this cannot be taken as evidence in support of the null hypothesis, because *p* values are unable to quantify support in favor of the null^50^. Therefore, Bayes Factors (BF) were calculated for group comparisons to assess the relative likelihood of the null and alternative (H1) hypotheses^51^. A BF_01_ of greater than 1 implies that the data are more likely to occur under H0 than under H1. Similarly, a BF_01_ lower than 1 indicates that the data are more likely to occur under H1 than under H0. Thus, if we analyze data and find that BF_01_ = 3, this means that the data are 3 times more likely to have occurred under H0 than under H1. Based on prior guidelines^52^, BFs between 1 and 3, between 3 and 10, and larger than 10 are interpreted as ambiguous, moderate, and strong support, respectively. Note that, where relevant, we provide natural logarithm values for Bayes Factors (log_e_(BF_01_)), which need to be exponentiated to get the BF_01_.

### Meta-analysis

Our exploratory individual difference studies were not designed to characterize a detailed pattern of associations between reasoning measures and intent-based moral judgment (e.g., some *specific* correlations would be stronger than others), but instead to firmly establish the *general* form of this association. Therefore, we carried out a random-effects meta-analysis^53,54^ using regression estimates (and the associated standard errors) across measures for each study and assessed if the meta-analytic effect was significantly different than 0. In addition to providing details from null hypothesis significance testing (NHST) approach, we also compute Bayes Factors for random-effects meta-analysis using default priors from *metaBMA* R package^55^.

### Data reporting

Statistical analysis was carried out in R programming language^56^ using *easystats*^57–60^ packages. For the sake of brevity, results from statistical analyses are included in the figures rather than the main text (an approach adopted in the R package *ggstatsplot*^61,62^). Similarly, details about demographics and experimental design for the studies are provided in Table 1.

### Ethics statement

Across all studies, participants provided written informed consent before any study procedure was initiated. Studies 1 and 2 were approved by the Ethics Committee of *Scuola Internazionale Superiore di Studi Avanzati* (Trieste) and the Hospital ‘Santa Maria della Misericordia’ (Udine), respectively. The Study 3 was approved by the Ethics Committee of Harvard University. All studies were conducted according to the principles in the Declaration of Helsinki.

#### Participants

See Table 1.

#### Measures

The following questionnaires were included across 3 studies (for more detailed descriptions, see Supplementary Text S1):

- *Need for Cognition* (NFC^63^) assesses the degree to which individuals are intrinsically motivated to engage in cognitive deliberation.
- *Cognitive Reflection Test* (6-item CRT^64^) captures people's ability to override an appealing but incorrect intuitive response.
- *Rational Experiential Inventory* (REI^65^) assesses the degree to which people engage in two modes of thinking: a fast, intuitive automatic thinking and a slower logical thinking.
- *Actively Open-Minded thinking* (AOT^66,67^) assesses individual differences in disposition to consider different conclusions even if they go against one’s own initial conclusion, to spend enough time on a problem before giving up, and to consider the opinions of others in forming one’s own opinions
- *Belief Bias* (BB^68^) measures the tendency to judge the strength of arguments based on the believability of their conclusion rather than how strongly they logically support that conclusion. Only syllogisms in which conclusions are logically invalid but believable (the class of problems that elicit high belief bias) are employed. They are taken from previous studies^67,69,70^.

## Results

**Figure 1:**
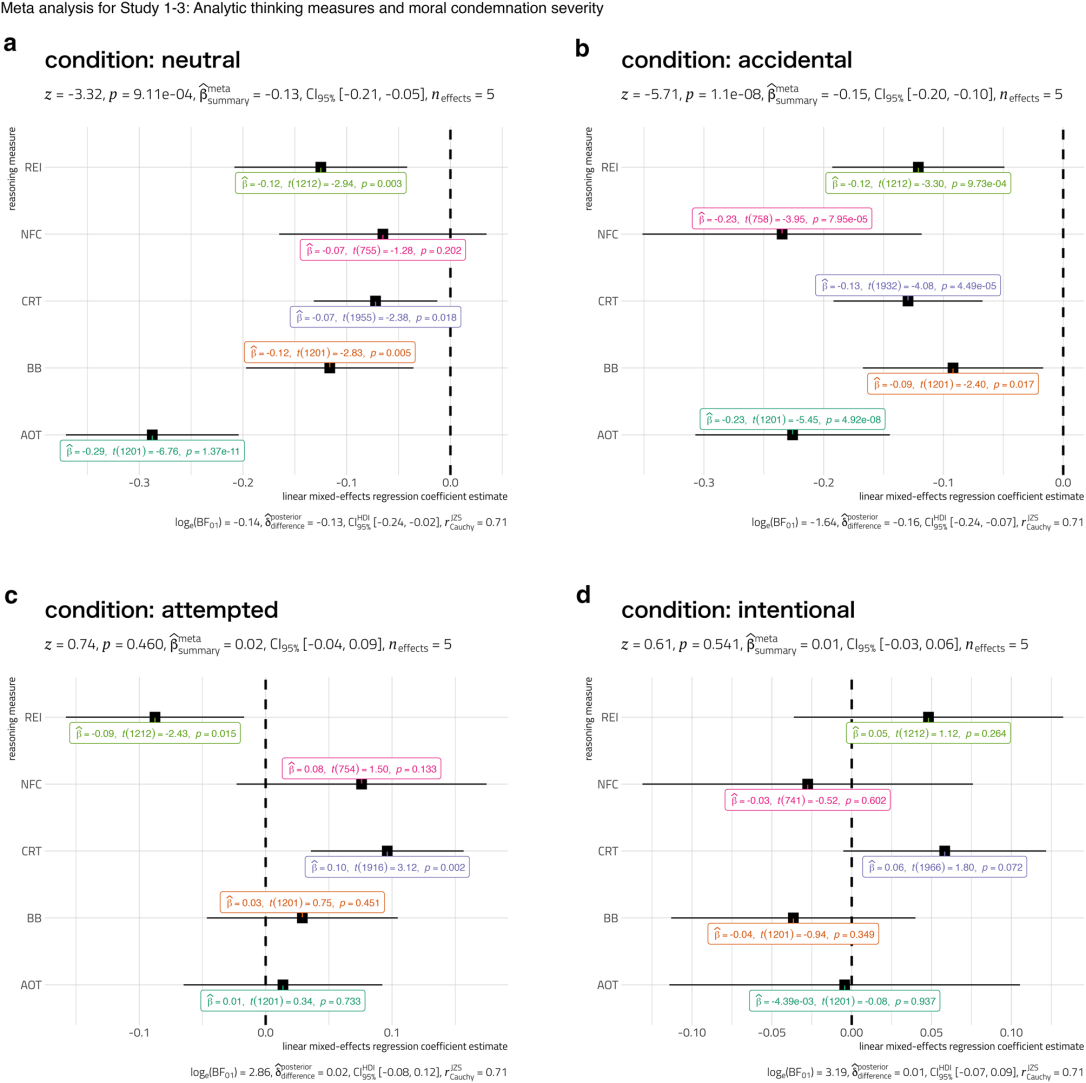
Regression coefficients for analytic thinking measures from linear mixed-effects regressions analyses carried out separately for each type of harm and each reasoning measure. The regression coefficient was significantly and consistently different from 0 across measures only for accidental harm condition. Error bars indicate 95% confidence intervals. Results from frequentist random-effects meta-analysis are shown in the subtitle, while results from Bayesian random-effects meta-analysis are shown in the caption. Although the meta-analytic effect is significant for neutral and accidental condition, Bayes Factor for the neutral condition reveals that the evidence in favor of the *alternative* hypothesis was inconclusive (BF_10_ = 1.15), while it was substantial for the accidental harm cases (BF_10_ = 5.15).

As mentioned before, our studies were not designed to explore reasoning measure-specific associations, but instead to establish the *general* form of this association. Accordingly, a random-effects meta-analysis of regression estimates revealed significant negative meta-analytic summary effect only for the neutral and accidental harm cases, such that people who scored higher on reasoning measures were more lenient in their assessment of such cases (see Fig. 1). But a more careful look at the Bayes Factor for the neutral condition reveals that the evidence in favor of the *alternative* hypothesis was inconclusive (BF_10_ = 1.15), while it was substantial for the accidental harm cases (BF_10_ = 5.15). Looking at Bayesian meta-analysis, we could also show that there was a strong evidence in favor of the *null* hypothesis for the attempted (BF_01_ = 17.46) and intentional (BF_01_ = 24.28) harm cases, i.e., summarizing across measures, there is no relationship between reasoning ability and tendency to judge attempted or intentional harm cases. Therefore, the meta-analysis supports our claim that scoring higher on reasoning measure is associated with greater tendency to judge third-party harmful transgressions more leniently, but only when the harm is caused accidentally.

## General discussion

Across a series of studies, we investigated the role that reasoning in determining the severity of moral judgments about harmful transgressions and observed that participants who self-reported to be more analytic and adept at cognitive deliberation by disposition were consistently more lenient in their judgments of accidental harms, as compared to participants who reported to rely more on the intuitive style of thinking.

The two-process model for intent-based morality^1,16^ argues that accidental harm produces a cognitive conflict between two processes: an agent-based, intent-driven response to forgive^10,11^ (based on innocent intentions) and a victim-based, empathy-driven impulse to condemn^9,14,71^ (based on harm caused). Current work expands on this work by focusing on the source of interindividual differences in how people resolve this conflict and assign blame or punishment to accidental harm-does and shows that analytical reasoning skills are one such source.

There are (at least) two possible ways in which reasoning can lead to a more lenient assessment of accidental harm-doers: (*i*) Individuals with better cognitive abilities also have more executive resources needed for Theory of Mind^72^, i.e. they are better at representing innocent mental states of the agent who accidentally harmed someone and thus forgive them. (*ii*) Individuals with higher propensity for cognitive deliberation are also better at down-regulating their empathic arousal stemming from harm appraisal and are thus more likely to forgive accidental harm-doers^71^. Future work should explore if it’s the cognitive (Theory of Mind) or the affective (empathic arousal) route or both that mediate the influence of reasoning ability on third-party moral evaluation.

## Limitations

Although our internal meta-analysis tries to draw conclusions that are generalizable across the battery of reasoning measures we utilized, the generalizability and robustness of these findings to a different set of reasoning and cognitive ability measures remains to be studied^73^. We hope future studies can overcome this limitation by employing a more comprehensive battery of cognitive measures. Additionally, the type of questions for moral judgement varied across the three studies (acceptability/wrongness; blame/punishment). Although the consistency of negative correlations between accidental harm and reasoning measures attests to the robustness of these effects to different framings, there needs to be a more systematic investigation about whether the *strength* of association might vary depending on the question asked^9,16^.

## Conclusion

Taken together, the present results argue that individual differences in reasoning are associated with differences in the way people cope with cognitive conflict when evaluating accidental harmful transgressions. The study of cognitive conflict (its detection and resolution) in the moral judgment field is an area of research still in its infancy, and we believe that the current work is a valuable addition to this growing field and hints at a number of exciting new avenue to explore.

## Supplementary Information

Supplementary Information.

## Data Availability

Data and analysis scripts are available from the Open Science Framework: https://osf.io/ayb7d/.
