# Supplementary material for: Reasoning supports forgiving accidental harms
Source: Sci Rep. 2021 Jul 13;11:14418. doi: 10.1038/s41598-021-93908-z (PMC8277901; doi:10.1038/s41598-021-93908-z)
Supplement: Supplementary file 1 — Supplementary Information. [file 41598_2021_93908_MOESM1_ESM.pdf]

Supplementary Information for-

"Reasoning supports forgiving accidental harms"

Supplementary Text S1: Detailed descriptions of *Methods* and *Results* sections

Study 1

Participants:

A total of 44 healthy people (29 female) were recruited and financially compensated for their time and travel. Average age was 23.01 years (SD = 3.07), with a range of 18 to 35. All participants gave written informed consent. This data was gathered during a larger fMRI study and the moral judgment task was completed in-scanner[1,2]. Although 50 participants were recruited for the fMRI study, only 44 completed the NFC questionnaire. This study was conducted according to the principles in the Declaration of Helsinki, approved by the Ethics Committee of the Hospital 'Santa Maria della Misericordia' (Udine, Italy), and was carried out in accordance with the approved guidelines. All participants provided written informed consent before any study procedure was initiated.

Material:

Need For Cognition: The 18-item short *Need for Cognition*[3] is a widely-used measure to assess the degree to which individuals are intrinsically motivated to engage in cognitive deliberation, i.e. how much they find thinking enjoyable and how frequently they engage in reflection without an external incentive to do so. The current study used the short 18-item NFC, which can be accessed from here: http://www.psy.ohio-state.edu/petty/documents/ncogscale.pdf

Participants reported agreement with statements (e.g., "I really enjoy a task that involves coming up with new solutions to problems.") on a 9-point Likert scale (-4: *very strong disagreement,* 0: *neither agreement nor disagreement*, +4: *very strong agreement*).

Scale for the moral judgment task: The experimental stimuli consisted of four different sets or versions of 36 unique vignettes for a total of 144 stories, adapted in Italian from a prior study[4], and participants were assigned different versions in Latin-square arrangement. After reading each story, participants provided two types of moral judgments[5,6], presented in randomized order across trials for each participant and presented on the screen for 6 s (this was part of fMRI timing specifications):

[1] *acceptability* - "How morally acceptable was [the agent]'s behavior?" (1: *Completely acceptable* to 7: *Not at all acceptable*);

[2] *blame* - "How much blame does [the agent] deserve?" (1: *None at all* to 7: *Very much*).

The intent-based vignettes were administered in a design with the following factors: belief (within-subjects: neutral, negative) × outcome (within-subjects: neutral, negative) × question (within-subjects: acceptability, blame).

Study 2:

Participants:

A total of 112 healthy native Italian-speaking community members (70 females) came to the lab to participate in this study and were financially compensated. All participants provided written informed consent. Average age was 24.12 (SD = 4.63) years, with a range of 18 to 50. All participants provided an informed consent before starting the study. This study was approved by the Ethics Committee of the Scuola Internazionale Superiore di Studi Avanzati (Trieste, Italy).

Material:

Cognitive Reflection Test consists of items featuring a problem which has an intuitively appealing answer that immediately comes to one's mind[7] but upon careful reflection turns out to be incorrect. For example, consider the following question:

If it takes 5 machines 5 minutes to make 5 widgets, how long would it take 100 machines to make 100 widgets?

If one were to rely solely on intuitive style of thinking, 100 minutes will be the most appealing answer, but reflective style of thinking entails engaging further in analytic processing and overriding this initial response to conclude that the correct answer is 5 minutes. Importantly, participants completed the CRT measure either before or after the moral judgment task, but it was one of the many questionnaires administered and thus the order effects were not relevant to the current study (cf. Paxton, Ungar, & Greene, 2012). We used the new psychometrically improved six-item version of CRT[9]. This questionnaire was administered among a battery of other questionnaires and thus we did not investigate the order effects of the administration of the questionnaire[8]. For each participant, CRT score was computed to be the sum of correct responses (score range: 0-6). We had predetermined to leave out any participant who didn't answer any one of the CRT items. No participant was excluded based on this criterion.

Scale for the moral judgment task: The experimental stimuli consisted of four different sets or versions of 36 unique vignettes for a total of 144 stories, adapted in Italian from a prior study[4], and participants were assigned different versions in Latin-square arrangement. After reading each scenario, participants provided two types of moral judgments[5] which were presented in a randomized order (for 6 s):

[1] *acceptability*: "How morally acceptable was [the agent]'s behavior?" (from "Not at all acceptable" to "Completely acceptable");

[2] *punishment*: "How much punishment does [the agent] deserve for his/her behavior?" (from "No punishment" to "The most severe punishment").

The intent-based vignettes were administered in a design with the following factors: belief (within-subjects: neutral, negative) × outcome (within-subjects: neutral, negative) × question (within-subjects: acceptability, punishment).

Participants responded using computerized visual analog scales (VAS), implemented as horizontal onscreen bar. A grey bar indicated the total range of possible responses, while a slightly narrower black asterisk included in the grey bar indicated the participants' current response. The position of the asterisk could be altered by using the "UP" and "DOWN" arrows on the keyboard. This scale was anchored with the aforementioned labels. Participants' responses were later converted to standardized scores with [min, max] of [0, 20]. The acceptability scores were reverse-scored so that higher score for the two questions indicated less acceptable behavior and more punishment.

Study 3

Participants: A total of 2435 US participants completed the task on Amazon Mechanical Turk for small financial compensation.

Method:

Participants were either administered rational component of Rational Experiential Inventory (REI) (*n* = 1223) or combination of Actively Open-Minded Thinking (AOT) and Belief Bias (BB) (*n* = 1212) measures:

(*i*) *Rational Ability* (RA), which indexes ability to think logically and analytically (e.g., "I have a logical mind."), and Rational Engagement (RE), which gauges reliance on and enjoyment of thinking in an analytical, logical manner e.g., "Thinking hard and for a long time about something gives me little satisfaction.", are subscales of Rational Experiential Inventory (REI)[10].

On the other hand, *Experiential Ability* (EA), which measures the ability with respect to one's intuitive impressions and feelings (e.g., “When it comes to trusting people, I can usually rely on my gut feelings”), and *Experiential Engagement* (EE), which assesses reliance on and enjoyment of feelings and intuitions in making decisions (e.g., “I often go by my instincts when deciding on a course of action”), compose the Experientiality component of REI.

Given that RA and RE were highly correlated (Pearson’s $r(1221) = 0.69$, $p < 0.001$) and rational items showed excellent internal reliability (Table 1), we created a single index for the rational component of REI. In its purpose, the Rational Experiential Inventory[10] was designed to assess the degree to which people engage in two modes of thinking: a fast, intuitive automatic thinking and a slower logical thinking. Theoretically motivated by Cognitive-Experiential Self-Theory or CEST[11], the various versions of the REI distinguish between two cognitive styles: (*i*) *a rational style*, measured by a modified version of Need for Cognition (NFC) scale[3,12] that emphasizes a conscious, analytical approach; (*ii*) an *experiential style*, measured by the Faith in Intuition (FI) scale, that emphasizes a pre-conscious, affective, holistic approach.

The REI scale is composed of 40 items. Participants judge to what extent the items describe them, using a 5-point ratings system (1 = *definitely not true of myself* to 5 = *definitely true of myself*). Following CEST theory, Epstein and colleagues[13] suggested the two processing modes to be independent of each other, so that it is possible to score high (or low) on both rationality and experientiality. For our purposes the REI-40 version[10] was used and only items corresponding to the rationality component were administered.

(*ii*) *Actively Open-Minded Thinking* scale[14,15] assesses the disposition to be fair towards different conclusions even if they go against one’s initially favored conclusion, to spend sufficient

time on a problem before giving up, and to consider carefully the opinions of others in forming one’s own. Participants answer the 8 items by using a 5-point scale ranging from "Completely disagree" to "Completely agree" with respect to each item proposed. It has been found to correlate with various other measures of reflective thinking, however, its original purpose was to show that the way people think is related to their beliefs about how they *should* think. Research by Stanovich *et al.*[16–18] found a reduced susceptibility, of AOT, to belief bias, probably related to the desire to be more informed before making an estimate or prediction.

Here the items of AOT scale:

1. Allowing oneself to be convinced by an opposing argument is a sign of good character.
2. People should take into consideration evidence that goes against their beliefs.
3. People should revise their beliefs in response to new information or evidence.
4. Changing your mind is a sign of weakness.
5. Intuition is the best guide in making decisions.
6. It is important to persevere in your beliefs even when evidence is brought to bear against them.
7. One should disregard evidence that conflicts with one’s established beliefs.
8. People should search actively for reasons why their beliefs might be wrong

(*iii*) *Belief Bias*[15,19] (BB) is the tendency to judge the strength of arguments based on the believability of their conclusion rather than how strongly they logically support that conclusion. Consequently, a person is more likely to accept arguments that support a conclusion that aligns with his/her beliefs and prior knowledge, while rejecting arguments in disagreement

with them. One context in which this phenomenon is particularly studied is that of syllogism reasoning[19–22].

A syllogism is a kind of logical argument that applies deductive reasoning to arrive at a conclusion based on two or more propositions that are asserted or assumed to be true. When the believability of the conclusion is incongruent with its logical validity (either believable but logically invalid, or unbelievable but logically valid), the syllogism is defined *conflict* syllogism. By contrast, syllogisms in which believability of the conclusions is congruent with its logical validity (either believable and valid, or unbelievable and invalid) are defined *control* (or *no conflict*) syllogisms; in this case, a response based on believability would be the same as a response based on logical validity. In the former case, when the conclusion following the premises is logically invalid but believable, individuals often produce *belief bias*, considering the conclusion as logically valid because it is congruent with their prior beliefs. Therefore, the tendency to produce belief bias is often explored by administering *conflict* syllogistic reasoning problems, and specifically logically invalid but believable syllogisms, the class of reasoning problems that elicit high belief bias. Therefore, for our purposes, only syllogisms in which conclusions are logically invalid but believable, are employed. They are taken from previous studies which employed them in the domain of morality[15] or from some of the major studies which explored *belief bias* in the context of syllogisms reasoning[19,23].

Below we provide the list of syllogistic problems employed:

Invalid, believable [15]

All flowers have petals.

Roses have petals.

Therefore, roses are flowers.

The conclusion logically follows the premises?

Invalid, believable [15]

All wives are married.

Some women are married.

Therefore, some women are wives.

The conclusion logically follows the premises?

Invalid, believable [15]

All things that have a motor need oil.

Automobiles need oil.

Therefore, automobiles have a motor.

The conclusion logically follows the premises?

Invalid, believable [23]

Not all highly trained dogs are vicious.

Some vicious dogs are police dogs.

Therefore, some highly trained dogs are not police dogs.

The conclusion logically follows the premises?

Invalid, believable [15]

All living things need water.

Roses need water.

Therefore, roses are living things.

The conclusion logically follows the premises?

Invalid, believable [19]

No addictive things are inexpensive.

Some cigarettes are inexpensive.

Therefore, some addictive things are not cigarettes.

The conclusion logically follows the premises?

Invalid, believable [19]

Some vitamin tablets are opprobines.

No nutritional things are opprobines.

Therefore, some nutritional things are not vitamin tablets.

The conclusion logically follows the premises?

Invalid, believable [19]

No well-educated people are Pennes.

Some judges are Pennes.

Therefore, some well-educated people are not judges.

The conclusion logically follows the premises?

Design: The intent-based vignettes were administered in a design with the following factors: belief (within-subjects: neutral, negative) × outcome (within-subjects: neutral, negative) × question (between-subjects: wrongness, punishment).

Supplementary Text S2: Detailed text of scenarios used

This section provides exact details of the vignettes used for the moral judgment task.

Study 1-2: Borrowed from a prior publication and reported therein[2].

Study 3: The four items used for this one study were the following four scenarios were used (different stems were combined to change levels of belief and outcome):

Chemical Plant/Coffee

Grace and her friend are taking a tour of a chemical plant. When Grace goes over to the coffee machine to pour some coffee, Grace's friend asks for some sugar in hers. There is white powder in a container by the coffee.
The white powder is just the regular sugar that the scientists use every day, and is therefore perfectly safe to have in coffee.
The white powder is a very toxic left behind by a scientist, and therefore deadly when ingested in any form.
The container is labeled "sugar", so Grace believes that the white powder by the coffee is sugar left out by the kitchen staff.
The container is labeled "toxic", so Grace believes that the white powder is toxic substance left behind by a scientist.
Grace puts the substance in her friend's coffee. Her friend drinks the coffee and is fine.
Grace puts the substance in her friend's coffee. Her friend drinks the coffee and starts vomiting blood.

Dentist

Frank is a dentist filling in the cavity of his patient. He must drill into the patients tooth just above a major nerve.
If the drill is used at a high speed, it will just dig the cavity and will not hit the nerve.
If the drill is used at a high speed, it will hit the nerve and cause excruciating pain.
Frank thinks that if he switches the drill to a higher speed he will dig the cavity without hitting the nerve below.
Frank thinks that if he switches the drill to a higher speed he will not only dig the cavity but also hit the nerve below.
Frank switches the drill to a higher speed, digs the cavity, and does not touch the nerve below.

Frank switches the drill to a higher speed, hits the nerve, and causes the patient excruciating pain.

Jellyfish/ Ocean

Irene and her neighbor are kayaking in a part of the ocean with lots of jellyfish. Irene's neighbor asks her if she should go for a swim.
It is perfectly safe to swim in the ocean, because the jellyfish do not sting and are totally harmless.
It is not safe to swim in the ocean, because the jellyfish sting and their stings are really painful.
Because Irene read information that said the ocean's jellyfish are harmless, she believes that it is quite safe to swim in the ocean.
Because Irene read information that said the ocean's jellyfish are harmful, she believes that it is not safe to swim in the ocean.
Irene tells her neighbor to go for a swim. Her neighbor does, enjoys the swim, and is just fine.
Irene tells her neighbor to go for a swim. Her neighbor does, gets stung by jellyfish, and experiences a lot of pain due to the sting.

Iron

Anna and her little sister are in the bathroom doing makeovers by the sink. Anna had straightened her hair earlier in the day using a straightening iron. The iron is still on the sink.
Anna's mother had turned the button on the iron off 3 h ago, so it is no longer hot and is perfectly safe to touch.
Anna's mother had just used the iron herself 5 min ago, so it is still extremely hot and could cause severe burns.
Because the cord on the iron was unplugged, Anna believes that it is no longer hot and cannot burn her sister.
Because the cord on the iron is still in the socket, Anna believes that it is still hot and could easily burn her sister.
Anna lets her sister continue to play by the sink. Her sister's arm hits the iron, but it is okay because the iron is cold. They have fun continuing their makeovers.
Anna lets her sister continue to play by the sink. Her sister's arm hits the iron, and she gets badly burned. She cries hysterically all the way the emergency room.

References

1. Patil, I., Calò, M., Fornasier, F., Young, L. & Silani, G. Neuroanatomical correlates of forgiving unintentional harms. *Sci. Rep.* **7**, 45967 (2017).
2. Patil, I., Calò, M., Fornasier, F., Cushman, F. & Silani, G. The behavioral and neural basis of empathic blame. *Sci. Rep.* **7**, 5200 (2017).
3. Cacioppo, J. T., Petty, R. E. & Kao, C. F. The efficient assessment of need for cognition. *J. Pers. Assess.* **48**, 306–7 (1984).
4. Young, L., Camprodon, J. A., Hauser, M., Pascual-Leone, A. & Saxe, R. Disruption of the right temporoparietal junction with transcranial magnetic stimulation reduces the role of beliefs in moral judgments. *Proc. Natl. Acad. Sci.* **107**, 6753–6758 (2010).

5. Cushman, F. Crime and punishment: distinguishing the roles of causal and intentional analyses in moral judgment. *Cognition* **108**, 353–80 (2008).
6. Patil, I., Young, L., Sinay, V. & Gleichgerrcht, E. Elevated moral condemnation of third-party violations in multiple sclerosis patients. *Soc. Neurosci.* **12**, 308–329 (2017).
7. Frederick, S. Cognitive Reflection and Decision Making. *J. Econ. Perspect.* **19**, 25–42 (2005).
8. Paxton, J. M., Ungar, L. & Greene, J. Reflection and reasoning in moral judgment. *Cogn. Sci.* **36**, 163–77 (2012).
9. Finucane, M. L. & Gullion, C. M. Developing a tool for measuring the decision-making competence of older adults. *Psychol. Aging* **25**, 271–88 (2010).
10. Pacini, R. & Epstein, S. The relation of rational and experiential information processing styles to personality, basic beliefs, and the ratio-bias phenomenon. *J. Pers. Soc. Psychol.* **76**, 972–87 (1999).
11. Epstein, S. The self-concept revisited: Or a theory of a theory. *Am. Psychol.* **28**, 404–416 (1973).
12. Cacioppo, J. T. & Petty, R. E. The need for cognition. *J. Pers. Soc. Psychol.* **42**, 116–131 (1982).
13. Epstein, S., Pacini, R., Denes-Raj, V. & Heier, H. Individual differences in intuitive-experiential and analytical-rational thinking styles. *J. Pers. Soc. Psychol.* **71**, 390–405 (1996).
14. Baron, J. Why Teach Thinking?-An Essay. *Appl. Psychol.* **42**, 191–214 (1993).
15. Baron, J., Scott, S., Fincher, K. & Emlen Metz, S. Why does the Cognitive Reflection Test (sometimes) predict utilitarian moral judgment (and other things)? *J. Appl. Res. Mem. Cogn.* **4**, 265–284 (2015).
16. Sá, W. C., West, R. F. & Stanovich, K. E. The domain specificity and generality of belief bias: Searching for a generalizable critical thinking skill. *J. Educ. Psychol.* **91**, 497–510 (1999).
17. Macpherson, R. & Stanovich, K. E. Cognitive ability, thinking dispositions, and instructional set as predictors of critical thinking. *Learn. Individ. Differ.* **17**, 115–127 (2007).
18. Stanovich, K. E. & West, R. F. Individual differences in rational thought. *J. Exp. Psychol. Gen.* **127**, 161–188 (1998).
19. Thompson, V. & Evans, J. S. B. T. Belief bias in informal reasoning. *Think. Reason.* **18**, 278–310 (2012).
20. Evans, J. S. B. T., Newstead, S. E., Allen, J. L. & Pollard, P. Debiasing by instruction: The case of belief bias. *Eur. J. Cogn. Psychol.* **6**, 263–285 (1994).
21. Evans, J. S. B. T., Barston, J. L. & Pollard, P. On the conflict between logic and belief in syllogistic reasoning. *Mem. Cognit.* **11**, 295–306 (1983).
22. Evans, J. S. B. T. & Curtis-Holmes, J. Rapid responding increases belief bias: Evidence for the dual-process theory of reasoning. *Think. Reason.* **11**, 382–389 (2005).
23. Morley (née Lambell), N. J., Evans, J. S. B. T. & Handley, S. J. Belief Bias and Figural Bias in Syllogistic Reasoning. *Q. J. Exp. Psychol. Sect. A* **57**, 666–692 (2004).
